# Microspheres give improved resolution in nondestructive examination of semiconductor devices

**DOI:** 10.1038/s41377-022-00747-2

**Published:** 2022-03-16

**Authors:** R. C. Woods

**Affiliations:** College of Engineering, University of South Alabama, Mobile, AL USA

**Keywords:** Super-resolution microscopy, Imaging and sensing, Spectrophotometry

## Abstract

The minimum spatial resolution of typical optical inspection systems used in the microelectronics industry is generally governed by the classical relations of Ernst Abbe. Kwon et al. show in a new Light: Science and Applications article that using an additional glass microsphere in the optical path can improve the resolution significantly.

## Abstract (web summary)

Artist’s impression (not to scale) showing a fanciful concept of how a glass microsphere produces a photonic nanojet that significantly improves resolution when examining microchip wafers with a microscope.

## Graphical abstract

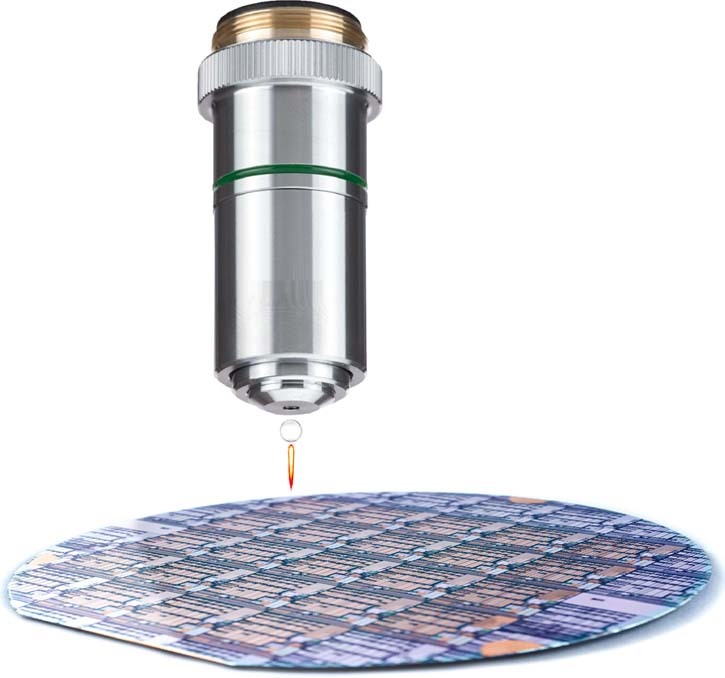

The first comprehensive theory of image formation in microscopes was formulated by Ernst Abbe in the 1870s and 1880s^1^. The basic principle conceived by Abbe was that a regular pattern of parallel lines used as a test object can be imaged and resolved as a pattern distinct from an unpatterned object if the first-order diffracted beams from the object are collected by the imaging objective lens. Abbe introduced the concept of “Numerical Aperture” (NA) to specify how far the objective lens was able to collect that diffraction pattern. The NA is the refractive index of the imaging medium (usually air, having refractive index close to unity) multiplied by the sine of the largest angle of light that can enter the objective lens; the simplest expression of the most important of Abbe’s results is that the smallest possible resolution is given by the optical wavelength divided by the NA. For well over a century this principle has formed the basis of the design of all microscopes and inspection and metrology equipment used in microelectronics and other fields.

Optical objectives having NAs on the order of unity are widely available from the major precision microscope manufacturers, but values larger than that are rare without using special immersion oil having a refractive index higher than unity. The use of immersion oil is impractical in microelectronics where the oil would contaminate the sample being inspected, though it is widely used in biological settings where a thin glass cover-slip is used to prevent oil contacting the object under scrutiny. This means that, in practice, in microelectronics and any other field where direct imaging is essential without using cover-slips, the smallest feature size examinable is roughly on the order of the wavelength of the light illumination used.

In recent years, the addition of glass microspheres to microscope optical paths has demonstrated the possibility of significant improvements in the resolution^2^. A microsphere is a sphere having a radius in the range 1 to 50 μm, typically made of an optical-grade transparent dielectric material^3^. There are several theoretical models that describe how microspheres overcome the traditional optical limits using white-light sources. Simplistically, the microsphere is placed much closer to the object than it is possible to place a conventional objective, and as a result, its effective NA is much larger because of its much larger subtended angle at the object. A transparent sphere behaves as a simple optical lens, though with considerable aberrations that are apparently insignificant compared to the resolution improvement. This is, however, an over-simplification; one of the most interesting models describing resolution enhancement based on microspheres is the photonic nanojet effect^4^. A photonic nanojet is an intense and extremely narrow light beam generated on the object side of the microsphere, by converting evanescent waves into propagating waves. The nanojet has width significantly smaller than would be expected using Abbe’s classical theory, enabling sub-Abbe resolution (or “super-resolution”) to be obtained. The exact mechanism of sub-Abbe resolution remains unknown, but super-resolution techniques using microsphere-assistance can be applied to diverse optical metrology systems such as interferometry and confocal microscopy^5,6^.

The new paper by Kwon et al.^7^ shows how these researchers were able to harness the super-resolution properties of a microsphere in a spectroscopic reflectometry system for microelectronics applications. Their paper is highly significant because previously such systems were limited by the Abbe criterion to examining features having minimum size roughly the same as the wavelength of the illuminating light, and in practice rather larger than that. A typical optical system uses white-light illumination at optical wavelengths of around 430–700 nm, and smaller features could traditionally not be examined. The authors used commercially available polystyrene or soda lime glass spheres to improve the resolution of their spectroscopic reflectometry equipment. They mounted a conventional objective lens (up to 100× magnification) on a piezoelectric actuator so that its position could be controlled automatically for the best results. Introducing the microsphere mounted on a micromanipulator so that its position could also be finely adjusted easily, they were able to use white light to examine features as small as 210 nm with additional magnification on the order of 5×. Since actual microscope objectives are imperfect, this compares to a resolution of around 1000 nm obtainable in practice using the conventional approach.

The authors also introduce a simple model of the effects of the microsphere, in which the added microsphere is represented by a thin optical lens additional to the conventional objective lens. This enables them to model how to adjust the position of the microsphere for optimum results, rather than using empirical guesswork.

In using the microsphere to assist their optical examination system, the authors show results for imaging particularly small features in microelectronic devices, such as commercial DRAMs, typically consisting of structures measuring 200 nm and even smaller. As well as imaging such structures optically using white light illumination, they were able to undertake spectroscopic reflectance measurements from individual parts of the DRAM structures, previously not possible without encroaching severely upon adjacent parts of the structure and thereby obtaining results corresponding to a “smeared-out” average of several adjacent parts of the DRAM. Conventional spectroscopic reflectance systems use a typical spot size of around 25 to 30 μm so that this breakthrough technique improves the spatial resolution of these measurements around 100-fold.
